# Covariates of diarrhoea among under-five children in India: Are they level dependent?

**DOI:** 10.1371/journal.pone.0221200

**Published:** 2019-08-21

**Authors:** Mala Ramanathan, Bevin Vijayan

**Affiliations:** Achutha Menon Centre for Health Science Studies, Sree Chitra Tirunal Institute for Medical Sciences and Technology, Trivandrum, Kerala, India; Institute of Economic Growth, INDIA

## Abstract

Program interventions like access to improved water supply, sanitation and hygiene do not have a systematic response to the aggregate health outcomes. Therefore, this is an attempt at recognising the concept of level sensitivity while verifying the association between prevalence of diarrhoea in under-five children in a district and its corresponding coverage of improved water supply and sanitation and hygiene. Information obtained in the DLHS—4 including 275 districts from 19 states and 2 union territories of India forms the database for this analysis. Universal access to safe drinking water, improving coverage of sanitation in a district beyond 71 percent across the country and beyond 78 percent among the non-south DLHS districts, has the potential to realise reductions in the prevalence of diarrhoea in under-five children in a district. The effect of improved sanitation seems to work synergistically with these indicators only at better levels of prevalence of diarrhoea in under-five children in a district. This offers lessons for the Clean India Mission in terms of universalising access to safe water and coverage up to three-fourths of households with sanitation in a district for the positive externalities to manifest in reduced prevalence of diarrhoea in under-five children.

## Introduction

Water, Sanitation and Hygiene (WaSH) are three basic dimensions of Public Health and any compromise in them has adverse consequences on health. Globally 71 percent of the people have access to safe drinking water in the premises, and 68 percent have access to basic sanitation services [1]. Hygiene constitutes many components like personal hygiene, hand washing, menstrual hygiene and food hygiene [1], and it is very difficult to measure the extent to which it is practised either by individuals or by groups. Maintaining good hygiene, however, remains crucial for preventing illness in general and for children in particular [2]. Hygiene is shaped by individual factors such as practices, beliefs, norms and educational status of caregivers of children and contextually by prevailing norms, e.g., access to safe drinking water and sanitation. Improvements in WaSH have an impact in reducing the incidence of diarrhoea and other intestinal infections. Availability and use of water along with hygiene practices reduces the risk of respiratory, skin and eye infections. Other benefits include the time saved in fetching water, preventing dropouts of girls from schools, improved attendance and long term gains in income [3]. Although improvements in WaSH on health have been widely recognised, they are hardly prioritised within the agenda of health programmes.

Diarrhoea and respiratory infections are two major causes of morbidity and mortality among children under five years of age in developing countries [4]. Deaths due to diarrhoea and pneumonia account for about 30 percent of the deaths in children worldwide and 90 percent of these deaths occur in sub- Saharan Africa and South Asia, with India accounting for about 28 percent [4,5]. India has the highest burden of diarrhoea in South Asia. It is an important public health problem as most of these deaths are preventable [6]. Globally water, sanitation and hygiene (WaSH) has contributed to a 13.4 percent reduction in the Disability Adjusted Life Years (DALYs) due to diarrhoea [7]. Major share of the disease burden is shared by the children in developing countries [3]. On this count, it is pertinent to take stock of the Indian situation wherein according to the estimates from the 69^th^ round of National Sample Survey Organisation (NSSO), 88.5 percent of households in rural areas and 95.3 percent in urban areas have access to an ‘improved source of drinking water’ (Bottled water, piped water into dwelling, piped water to yard/plot, public tap/stand pipe. Tube well/borehole, protected well, protected spring, and rainwater collection). Similarly, the coverage of ‘improved source of sanitation’ in rural and urban areas was 38.8 percent and 89.6 percent respectively [8]. Spatial and temporal analysis using DLHS 3 and NFHS 4 data indicate clustering of diarrhoea cases within and across districts of India [9].

Preventive strategies for diarrhoeal diseases have centred around promoting exclusive breastfeeding and personal hygiene at an individual level and immunization specific to diseases like rotavirus, cholera and improving water and sanitation at a community level [10]. In recent times, there is an increased push towards introduction of rotavirus vaccines for the prevention of diarrhoea in children. It has been argued that sanitation and hygiene improvements have less impact on rotavirus disease and hence the need for vaccination to prevent it. On the contrary, diarrhoeal diseases in industrial countries came down even in the absence of rotavirus vaccines highlighting the role of improvements in sanitation and hygiene [11,12]. Coverage through immunisation requires an effort by the health system whereas WaSH initiatives need a multi-sectoral approach. The persistent prevalence of diarrhoea as a major cause of death among children calls for examining the varying potential of interventions to bring it down.

The District Level Health Surveys offers the relevant information-base to verify the possible association between the prevalence of diarrhoea and the key WaSH indicators of safe drinking water and sanitation facilities. Hygiene, while being a key component of WaSH is far more difficult to measure as it involves multiple actions routinely performed by caregivers of children and others to maintain health. One can seek to capture the propensity to maintain appropriate hygiene in care through a proximate variable. Maintaining hygiene standards involves both knowledge of ***how to act*** and the ***means to act***. Therefore, literacy may serve to represent this–that is the knowledge and comprehension of the need for appropriate hygiene practices. Further, female literacy, in particular, becomes more appropriate as much of childcare is provided by women and may serve as a proxy for the hygiene component of WaSH.

### Relevance of level sensitivity

The association between prevalence of diarrhoea may not hold uniformly across the range of coverage of safe water supply, sanitation or extent of female literacy across regions. In addition, improved coverage of water supply and sanitation can have a synergistic effect on behaviour and practices with regard to childcare and thus influence the prevalence of diarrhoea. At uniformly high or low levels of safe water or sanitary conditions, one cannot expect a uniform impact on diarrhoea prevalence. Such effects would manifest themselves at specific thresholds when synergistic mechanisms operate. Therefore, it is pertinent to recognise varying threshold levels at which these variables gain salience to affect the prevalence of diarrhoea in a district or across regions. Identifying these thresholds may help to determine the push needed with respect to ‘Clean India Mission’ or ‘Swachh Bharat Mission’ that is a flagship mission of the Govt of India. This programme aims to achieve universal sanitation coverage by 2019. Two key objectives of the Swachh Bharat Mission focus on bringing about improvements in the general quality of life in rural areas by promoting cleanliness, hygiene and elimination of open defection and also to accelerate sanitation coverage in rural areas [13].

Temporal or cross-sectional examination of associations between development indicators tends to value a quantum of change uniformly across the entire range of values that indicators assume. However, while considering success indicators like immunization rates or antenatal care coverage rates; or failure indicators like infant or child mortality or morbidity rates; efforts needed to realise a change in the indicator at better levels need to be valued more when compared with such a change at worse levels [14]. Increasing immunization coverage in a district from 80% to 90% needs greater effort when compared to the effort needed to shift it from 40% to 50%. Similarly, for a failure indicator like Infant Mortality Rate, a shift of three units from 16 to 13 calls for greater effort than shifts from 45 to 42 [15]. This differential valuation is referred to as level sensitivity [16] and attributes greater value to the same quantum of change in an indicator at better levels when compared to the same quantum at less than better levels [17]. While universal sanitation coverage may be a desired goal, its externalities on under-five child health may be experienced at lower levels of coverage. Given the efforts that may be needed to achieve sufficient coverage of the various WaSH indicators such as sanitation coverage and percentage of households covered by safe drinking water facilities within the district to have an impact on child health, identifying the thresholds at which these externalities are achieved may provide inputs for the Swachh Bharat Mission.

The outcome of interest in this analysis is a failure indicator—diarrhoeal prevalence among children under five years of age. We examine the role of coverage of safe drinking water, improved sanitary conditions and appropriate hygiene practices by mothers and their association with diarrhoeal prevalence in a district. We include the indicator of female literacy namely, the percentage of women with education beyond high school in a district, as a proxy to depict improved hygiene practices adopted by mothers. Recognition of the concept of level sensitivity in this context is useful as it would help to identify the threshold or level where the relationship of these household level infrastructural indicators gains salience with respect to association with the outcome of interest–viz. diarrhoeal prevalence among children under five years of age. Identification of these threshold levels can serve as a guide to shape the required intensity of the interventions. The analysis proposes to determine the threshold levels at which improved water supply and improved sanitary conditions and hygiene practices by mother’s affect prevalence of diarrhoea among under-five children. By this, we mean, that the analysis aims to identify the levels at which these indicators become sensitive to variations in the prevalence of diarrhoea in under-five children across districts.

## Methods

The District Level Household Survey-4 (DLHS-4) is a demographic and health survey at the district level conducted by the Ministry of Health and Family Welfare in India. It provides information on 275 districts from 19 of the 29 states and 2 of the 7 union territories (UT’s) in India for the period 2012–2013 [18]. The fact sheets of these 19 states and 2 UT’s contained information on 2,97,310 women. ‘Coverage of safe drinking water’ in the DLHS-4 survey has been conceptualised as ‘having improved source of drinking water’(which included piped water into dwelling, piped to yard or plot, public tap or standpipe or hand pump or tube well or bore-well or well covered or protected spring or tanker or truck or cart with small tank or drum and packaged bottled water). Improved sanitation has been conceptualised as ‘having access to improved toilet facilities’(which included flush to sewer/septic pit, pit with slab, pit ventilated improved and other improved modes). Improved female literacy has been defined as ‘percentage of currently married women with 10 or more years of schooling’. We have used this, instead of ‘female literacy’ as a behavioural change in terms of use of improved sources of drinking water and sanitation would need sufficient years of education to overcome existing norms and practices. The DLHS-4 reports prevalence of diarrhoea during the last two weeks among under-five children in the household and this indicator is computed at the district level against which the WaSH predictors are examined. The data were analysed using SPSS version 17 [19], and the graphs were developed using ggplot2 package in R [20,21].

## Statistical analysis

The districts were grouped by level of the outcome variable, i.e. prevalence of diarrhoea in under-five children, in terms of 20th, 40th, 60th and 80th percentiles in descending order as the outcome of interest is a failure indicator. These points are represented by 6.7% (80th percentile and above), 5.04% (60th percentile and above), 3.66% (40th percentile and above) and 2.3% for the lowest prevalence of diarrhoea in under-five children (20th percentile and above). The relationship between the outcome variable, i.e. prevalence of diarrhoea in under-five children in the district with safe water supply, improved sanitation and female literacy were examined within each of the percentile groups using OLS regression. OLS has been preferred here instead of Quantile regression as the purpose is to examine the changing effect of the predictors across the cumulative spectrum of the variation in the outcome variable- viz prevalence of diarrhoea in the under five.

**Fig 1:**
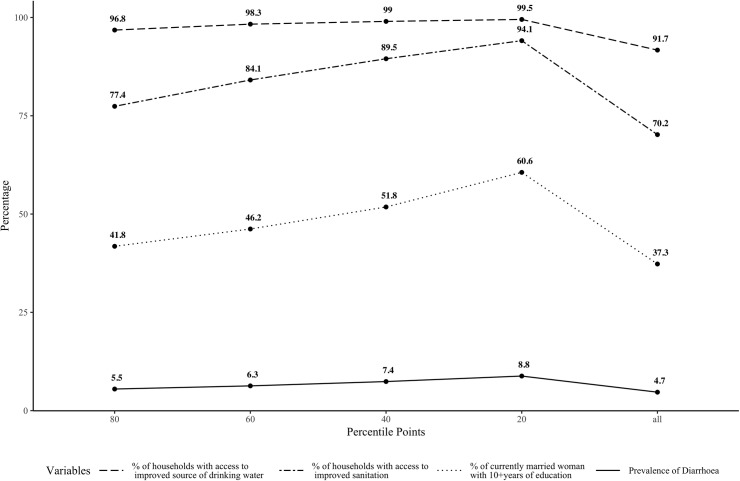
Mean of Diarrhoea Prevalence and other specific WaSH predictors (including districts upto and above 80^th^, 60^th^, 40^th^ and 20^th^ percentile points), DLHS-4 states, 2012-'13.

Fig 1 shows the mean of the outcome and predictor variables upto the 20^th^, 40^th^, 60^th^ and 80^th^ percentile along with their overall mean to depict the distribution of the mean across each percentile.

The means of the outcome variable, prevalence of diarrhoea in under-five children and the predictor variables tends to shift with the inclusion of the data points from the five cut-offs identified along the range with a clear convergence to the mean. However, at none of the levels, is there equivalence to the overall mean. That all the data points along the range converge to the mean at different rates is visible from Fig 1 –indicating that the variances are not uniform. The distribution of the outcome and predictor variables indicates that the convergence to the mean is not uniform through the 20 percentile points. The outcome variable, i.e. prevalence of diarrhoea in under-five children has a linear trajectory in terms of the convergence of the mean as does the dependent variable, improved sanitation. What is discernible is that the change in the mean across districts for the percentage of households with access to improved sanitation and percentage of households in a district with women who had more than 10 years of education has a rather steep gradient with the inclusion of additional districts along the percentile points. This indicates that with respect to these two indicators there is a sharp change in the variation across the range contrary to the assumption of uniform variance for analysis using Ordinary Least Squares (OLS) regression. This assumption holds approximately, only for one of the WaSH variables, percentage of households with access to improved drinking water, and only up to the 20th percentile point. We have tested the predictor variables for all the cut-offs used. The Shapiro-Wilks W was significant for the analysis above 80th, 60th, 40th, 20th and including the 275 districts as a whole. The normality assumption does not hold for these WaSH variables considered. This will bias the estimate of the standard error of regression coefficients to some extent [22]. However, as the linearity assumption holds to some extent, we have used OLS Regression for this analysis as it has been found valid when samples sizes are large [23]. This means that change in each of the indicators has varying effect across the range of the outcome of interest, viz, prevalence of diarrhoea in under-five children in a district across all districts. This is the rationale for the use of level sensitivity analysis to examine the association between the levels of diarrhoea and the identified WaSH predictor variables.

## Results and discussion

The specific distributions of 275 districts by the outcome and predictor variables are described in Table 1.

**Table 1:** Distribution of districts by Diarrhoea Prevalence and selected WaSH predictors^a^, DLHS-4 states, 2012-'13.

Descriptives	Coverage of improved drinking water	Coverage of improved sanitation	Currently married woman with 10+ years of schooling	Prevalence of diarrhoea
Percentile point				
Number of districts	275	275	275	273
20^th^	87.4	51.04	24.90	2.3
40^th^	95.58	65.84	31.96	3.66
60^th^	97.8	79.66	38.12	5.04
80^th^	99	88.98	48.46	6.7
Mean (of coverage in %)	91.7	70.3	37.3	4.7
Median (of coverage in %)	96.9	74.3	35.0	4.3
Range (of coverage in %)	32.3–100.0	17.5–99.8	6.9–93.8	0.0–22.2

^a^predictors- Women with 10+ years of schooling, coverage of improved drinking water and coverage of improved sanitation

Coverage of safe drinking water across the districts ranged from a low of 32.3% to universal coverage (100%). The coverage of improved sanitation across districts varied within a wide range of 17.5% to near-universal coverage (99.8%). Similarly, female literacy across the 275 districts had a range of 86.9 percentage points. The prevalence of diarrhoea in under-five children during the past two weeks across districts went from near non-existent (0.0%) to almost one-fourth of the children (22.2%).We used OLS regression to assess the degree of association between the predictor variables and the outcome. The predictive efficacy (adjusted R^2^) is not high, which is expected given that the contextual associations in cross-sectional data are always marginal. However, the associations are strong, and that in itself is sufficient to determine the thresholds before or after which the associations hold. OLS regression of the outcome variable–prevalence of diarrhoea in under-five children, with the predictors are described at each of the 20^th^ percentile points to examine the strength of the associations is shown in Table 2.

**Table 2:** Association between selected WaSH predictors^a^ and Diarrhoea Prevalence by specific percentile-points, DLHS-4 states, 2012-'13.

Type of inclusion (n)	R^2^ (p value)	Coefficient β (p value)		
		Currently married woman with 10+ years of schooling	Improved sanitation	Improved drinking water
All	0.08 (<0.01)	0.033 (0.01)	-0.046 (<0.01)	-0.027 (0.05)
Above 80^th^ (≥6.7)	0.006 (0.96)	-0.02 (0.59)	0.004 (0.87)	0.006 (0.82)
Above 60^th^ (≥4.6)	0.012 (0.74)	0.003 (0.91)	-0.008 (0.61)	-0.019 (0.33)
Above 40^th^ (≥3.2)	0.038 (0.1)	0.018 (0.31)	-0.025 (0.03)	-0.028 (0.08)
Above 20^th^ (≥2)	0.079 (<0.01)	0.037 (0.01)	-0.04 (<0.01)	-0.029 (0.04)

^a^ predictors- Women with 10+ years of schooling, coverage of improved drinking water and coverage of improved sanitation

The OLS regression using all the 275 districts (Adjusted R^2^ = 0.07) indicated that improved sanitation (β = -0.046, p<0.01), improved drinking water (β = -0.027, p = 0.05) and female literacy (β = 0.033, p = 0.01) were significant predictors of the prevalence of diarrhoea in under-five children. We need to understand if this association hold uniformly across all levels of diarrhoea prevalence in under-five children or does it vary by its level? Conditioning the analysis by levels of prevalence of diarrhoea in under-five shows that improved sanitation has an effect only beyond the 40^th^ percentile point while female literacy and improved drinking water gain salience above the 20^th^ percentile point.

We noticed some clustering within the predictor variables by region. The districts from the southern parts of India have very high levels of improved drinking water supply and good sanitation, and this contributes to increased variance in the overall analysis. The distribution of the means across the percentile points for diarrhoea in under-five children for the southern districts and other non-south districts is shown in S1 Table. To examine this feature, we stratified the districts by region and used the same model for OLS regression for southern and other districts. These results are provided in Table 3.

**Table 3:** Ordinary least squares regression of prevalence of diarrhoea in under-five children by regions, 2012-'13.

Type	R^2^ (p value)	Coefficient β (p value)		
		Currently married woman with 10+ years of schooling	Improved sanitation	Improved drinking water
South	0.09 (0.02)	-0.035 (0.12)	-0.01 (0.5)	-0.007 (0.81)
Other Districts	0.11 (<0.01)	0.064 (<0.01)	-0.05 (<0.01)	-0.038 (0.02)

None of the three predictors are strongly associated with the outcome of interest when we consider only the southern districts, which comprised of districts from Tamil Nadu, Kerala, Karnataka, Telangana, Andhra Pradesh, Puducherry and Andaman and Nicobar Islands. This is because the range of the values for outcome variable is minimal in this restricted data set.

Most of the variation in the outcome of interest is explained by variation in the predictor variables in the non-south districts of India. For this reason, we have repeated the level sensitivity analysis for the non-south districts, which consisted of 168 districts in all and these results are shown in Table 4. Surprisingly, the cut-offs for the dependent variable, viz. Prevalence of diarrhoea among under-five children remained the same for this sub-group as well.

**Table 4:** Association between selected WaSH predictors^a^ and Diarrhoea Prevalence by specific percentile-points, DLHS-4 non-south States, 2012-'13.

Type	R^2^ (p value)	Coefficient β (p value)		
		Currently married woman with 10+ years of schooling	Improved sanitation	Improved drinking water
Above 80^th^ (≥6.7)	0.026 (0.84)	-0.02 (0.69)	-0.021 (0.59)	0.01 (0.58)
Above 60^th^ (≥4.6)	0.049 (0.35)	0.045 (0.15)	-0.03 (0.17)	-0.02 (0.25)
Above 40^th^ (≥3.2)	0.09 (0.02)	0.055 (0.017)	-0.035 (0.04)	-0.046 (0.03)
Above 20^th^ (≥2)	0.109 (<0.01)	0.059 (0.03)	-0.06 (<0.01)	-0.03 (0.08)

^a^ predictors- Women with 10+ years of schooling, coverage of improved drinking water and coverage of improved sanitation

The extent of improved drinking water available in a district gains salience only when the 40^th^ percentile point and all those above it are included. This then marks the threshold where improved drinking water availability is significantly associated with the outcome of prevalence of diarrhoea in under-five children. Examining the distribution of extent of availability of improved drinking water, we find that at this threshold the availability ranges between 32.3 percent to 100 percent with a median of 96 percent. Clearly for the level of availability of improved drinking water to gain salience with respect to the prevalence of diarrhoea in under-five children, near-universal coverage is needed.

Improved sanitary facility is associated with the prevalence of diarrhoea in under-five children only above the 40th percentile point of the distribution prevalence of diarrhoea across the districts. The median of improved sanitary facilities is 77.7 percent coverage of sanitation across districts. This means at the very least close to three fourths or more of the households in the district need to have improved sanitary facilities before one can see a reduction in the prevalence of diarrhoea in under-five children in a district.

The potential to use hygiene practices by mothers, measured by the proportion of married women with 10+ years of education affects the prevalence of diarrhoea in the districts only below the 40th percentile point of the district-wise distribution of prevalence of diarrhoea. This association is however not as expected. Higher literacy is associated with higher prevalence of diarrhoea. An examination of the relationship indicates the deviation from the expected at the mid-region contributes to this aberration, and beyond this central point, the association with the prevalence of diarrhoea is rendered meaningful. The median level of literacy of currently married women for this set is 36.4 percent with a range of 6.9 to 66.4 percent.

We set out to examine the relationship between availability of household level WaSH indicators like improved drinking water, improved sanitation and potential to use hygiene-related practices on health outcome viz prevalence of diarrhoea in the under-five children. It is apparent that these variables do have an effect on the prevalence of diarrhoea in under-five children across the districts. Given that the districts in the south have better infrastructure within the households in terms of access to improved drinking water and improved sanitation when compared to the other districts, a stratified analysis by regions is attempted. This revealed that these variables did not have any effect on the prevalence of diarrhoea in the southern districts but had an effect on the prevalence in districts elsewhere.

Such generic findings have little or no implication from a policy perspective. Nevertheless, it undoubtedly hints at interventions with a differential emphasis in a high diarrhoea prevalence district as against the low prevalence one. Such differential emphasis needs to be guided by the dynamics of relationship at different levels of prevalence. To this end, we employed the concept of level sensitivity as described by Mishra and Subramaniam (2006) where the relationships between the outcome and predictor variables were conditioned by the level of the prevalence of diarrhoea in under-five children. Level sensitivity analysis was undertaken by including all districts of India for which reports are available in the DLHS-4, indicate that improved toilet facilities and improved drinking water have no effect at very high levels of prevalence of diarrhoea. However, initial declines were noticed with a higher level of access to improved drinking water and improved sanitation facilities. At lower levels of prevalence of diarrhoea, the salient association is with improved female literacy.

## Conclusions

This analysis is not without limitations which include low explanatory capacity of the predictor variables in the regression model as indicated by the low R square values. In addition, even though the predictor variables and the examined outcome are more or less linear, the departures from normality may provide biased regression coefficients. Nevertheless, the results indicate a need to focus on WaSH indicators to bring about positive externalities on child health. While prioritizing among multiple interventions that have a bearing on an outcome, the level of the outcome should guide such prioritization. This exercise offers an explanation of the three components namely; access to improved water supply, improved sanitation and female education gain statistical salience or significance, conditioned by the level of outcome of prevalence of diarrhoea in under-five children. For instance, it is observed that improved sanitation has an effect only beyond the 40th percentile point of levels of diarrhoea prevalence in a district while female literacy and access to improved water supply gain salience above the 20^th^ percentile point. This exploration indicates that it may be possible to reduce diarrhoea prevalence in the under-five children by universalising access to improved drinking water and attempting to improve access to sanitary facilities in the district to more than 71 percent of the households within a district n general. But for the non-southern states, improved sanitary facilities will need to be accessible to at least 78 percent of the households in a district to affect the prevalence of diarrhoea in under-five children. Hence recognition of the level of prevalence of diarrhoea in under-five children in a district should guide the prioritization among the various intervention options such as improved water supply or sanitation of increase in proportion of women with higher levels of education. There is a message for public policy in terms of the Swachh Bharat Mission, that the positive externalities of universal sanitation mission, when teamed with synergistic effects of access to safe drinking water and educated women within a district may have health dividends in terms of the reduced prevalence of diarrhoea in under-five children.

## Supporting information

S1 Table Mean distribution of specific predictors* by percentile-points for Diarrhoea Prevalence, DLHS-4 and non-south districts, 2012-'13. (PDF)
