# Supplementary material for: Covariates of diarrhoea among under-five children in India: Are they level dependent?
Source: PLoS One. 2019 Aug 21;14(8):e0221200. doi: 10.1371/journal.pone.0221200 (PMC6703849; doi:10.1371/journal.pone.0221200)
Supplement: S1 Table — (PDF) [file pone.0221200.s001.pdf]

Covariates of Diarrhoea Among Under-five Children in India: Are they level dependent?

Supplementary Table S1 Mean distribution of specific predictors* by percentile-points for diarrhea prevalence, DLHS-4 and non-south districts, 2012-'13

Prevalence of diarrhoea	Descriptives	Coverage of improved drinking water	Coverage of improved sanitation	Currently married woman with 10+ years of schooling
		**DLHS-4 districts**		
Above 80th:≥ 6.7	mean	89.12	64.36	38.57
n= 57	SE of mean	2.22	2.46	1.76
Above 60th:≥ 5.04	mean	91.02	64.41	38.17
n= 109	SE of mean	1.32	1.77	1.17
Above 40th:≥ 3.66	mean	91.49	60.78	37.95
n= 164	SE of mean	1	1.5	0.99
Above 20th:≥ 2.3	mean	91.47	68.98	37.29
n= 221	SE of mean	0.87	1.31	0.93
		**DLHS-4, non- south districts**		
Above 80th:≥ 6.7	mean	84.89	70.87	39.83
n= 35	SE of mean	3.41	2.82	2.58
Above 60th:≥ 4.6	mean	87.68	71.48	37.52
n= 109	SE of mean	1.99	2.05	1.65
Above 40th:≥ 3.2	mean	89.22	73.14	36.79
n= 164	SE of mean	1.45	1.69	1.41
Above 20th:≥ 2	mean	88.84	75.63	38.2
n= 221	SE of mean	1.29	1.37	1.18

*predictors- Women with 10+ years of schooling, coverage of improved drinking water and coverage of improved sanitation
